# Reply to Landwehr, K.R.; Larcombe, A.N. Comment on “Karthikeyan et al. Concordance between In Vitro and In Vivo Relative Toxic Potencies of Diesel Exhaust Particles from Different Biodiesel Blends. *Toxics* 2024, *12*, 290”

**DOI:** 10.3390/toxics13030186

**Published:** 2025-03-05

**Authors:** Subramanian Karthikeyan, Dalibor Breznan, Errol M. Thomson, Erica Blais, Premkumari Kumarathasan

**Affiliations:** 1 Environmental Health Science and Research Bureau, Health Canada, 251, Sir Frederick Banting Driveway, Ottawa, ON K1A 0K9, Canada; dalibor.breznan@hc-sc.gc.ca (D.B.); errol.thomson@hc-sc.gc.ca (E.M.T.); erica.blais@hc-sc.gc.ca (E.B.); 2 Department of Biochemistry, Microbiology & Immunology, Faculty of Medicine, University of Ottawa, Ottawa, ON K1H 8M5, Canada; 3 Interdisciplinary School of Health Sciences, Faculty of Health Sciences, University of Ottawa, Ottawa, ON K1N 6N5, Canada

This reply aims to address the comments made by Landwehr and Larcombe [1] regarding our article “Concordance between In Vitro and In Vivo Relative Toxic Potencies of Diesel Exhaust Particles from Different Biodiesel Blends. *Toxics* **2024**, *12*(4), 290” [2].

We appreciate the acknowledgement by Landwehr and Larcombe of the paucity of biodiesel toxicology studies, the complexity of emission toxicity testing using in vitro and in vivo platforms, and the challenges inherent to linking in vitro and in vivo outcomes. We are also thankful for their acknowledgement of our effort in conducting this work: “the authors should be commended for expanding the knowledge on biodiesel exhaust health effects, particularly in their comparison of different types of biodiesel within the same study when many previous articles assess only one type and make general assumptions about biodiesel as a whole based on their findings”.

In order to address the toxicity and health impacts of ambient air pollutants—of which traffic-related air pollution (TRAP) is an important contributor—it is essential to understand the toxicity of individual components as well as mixtures. In our paper, the focus was on gaining an understanding of the relative toxicities of particulate fractions of diesel emissions from the combustion of various diesel blends, and the questions were posed and addressed accordingly. The comments of Landwehr and Larcombe pointing to potential gaps or limitations in this study did not take into consideration some important nuances relevant to the assessment of particle toxicity. Below, we provide point-by-point responses to the issues raised in Landwehr and Larcombe’s comments.

In writing the paper, we strived to cite the published work most relevant to our study rationale and design, and to the interpretation of our findings. We note that the studies identified by Landwehr and Larcombe [3,4,5,6,7] (as examples of work reporting biodiesel toxicity rankings that apparently ‘conflict’ with our findings are not directly comparable to our work owing to several reasons, most notably the distinct respective goals of each study. Specifically, while our work compared the relative toxicity of emission-derived particles, a key exhaust constituent implicated in adverse health effects, Landwehr et al. 2021 and 2023 assessed the relative toxicity of the whole exhaust; Andre et al. (2015) examined the mutagenicity and genotoxicity of diesel PM and the whole exhaust; and Jetton et al. (2024) studied metabolic effects in the offspring of mice after exposure of the mice to diesel PM. Landwehr et al. 2021 and Landwehr et al. 2023 used in vitro exposures at the air–liquid interface and in vivo exposures through inhalation, respectively, in an effort to understand whole-exhaust toxicity, while we used in vitro exposures of submerged cell cultures and in vivo exposures conducted via the intratracheal instillation of particle suspensions in order to delineate and compare the toxicity of particulate emissions. Any direct comparison of our findings to those based on the papers cited by Landwehr and Larcombe will be complicated by differences in the engine and emission treatment technology used, animal or cell model chosen, exposure methodology, dose and duration of exposures, and endpoints measured. Ultimately, models and approaches are selected in relation to the objective, which, in our study, was to assess PM toxicity. For our study purpose, whole-exhaust exposures were not appropriate, as the effects of PM cannot be readily disentangled from the effects of gases. Therefore, we suggest that the criticism presented regarding “conflicts with multiple studies” fails to recognise the significant differences in the approaches of the studies cited by Landwehr and Larcombe as examples.

Landwehr and Larcombe highlight the importance of using “real-world relevant exposure methodology”. Understanding the effects of the entire pollutant mix, which includes gaseous and volatile components, is indeed an important aspect of toxicological investigation into potential health outcomes. We are aware of the value of conducting in vivo [8] and in vitro [9] experiments on airborne exposures to freshly generated emissions. However, we would argue that the selection of the methodology should be driven by the purpose of the study. There are several important benefits of evaluating the effects of specific constituents, such as particulate matter. These include, notably, the capacity to link biological effects to PM rather than to the emission as a whole, providing insight into determinants of toxicity in support of regulatory efforts. Furthermore, the collection of PM and parallel exposure of cells enables a direct comparison of effects under identical conditions. Archived material can be compared at future dates against other test materials, enabling, for example, an evaluation of temporal and spatial variability in PM toxicity [10,11], an approach that can be applied to examining the effects on toxicity from changes in engine technology or other factors over time. It should also be noted that exposures to whole emissions have their own limitations. These include, but are not limited to, the challenge of disentangling the effects of individual constituents; the inability to directly compare, under equivalent conditions, emissions generated at different times; and the question of accounting for the atmospheric transformation of the air pollutants. Ultimately it is by using a variety of approaches—each with its strengths and limitations—selected on the basis of relevance to the question of interest, that we gain a greater understanding of the determinants of toxicity.

We agree that emission treatment technology and standards have evolved over the past several years. Indeed, this was highlighted in our paper: “Generally, new heavy-duty on-road engines, along with advanced combustion, fueling, and thermal management strategies, are equipped with DPFs and SCR (selective catalyst reduction) systems to meet more stringent emission standards. Future emission standards are based on varying test conditions and are approaching 0.02 g/bhp-hr for NOx and 0.005 g/bhp-hr for PM”. Nevertheless, we would argue that the findings from this work using 2004 certified heavy-duty engine technology are still relevant, as the resultant toxicity contrasts enable the generation of hypotheses regarding toxicity determinants among emission particles of different feedstock origins. As PM regulations continue to be based on mass, rather than composition, evidence supporting the notion that composition, too, drives potency remains relevant.

The filters used in the work are Zefluor^®^ 8 “×10” rectangular polytetrafluoroethylene (PTFE) filters (Pall Life Sciences, Port Washington, NY, USA) with a pore size of 0.2 µm (200 nm). Particles collected using Zefluor filters undergo a number of processing steps before use for exposures. Initially, the filter is sonicated in water to extract particles (a standard approach). The particle extracts obtained this way are then lyophilized and stored. Prior to exposure, the lyophilized particles are suspended in a particle preparation buffer, vortexed, and sonicated to obtain well-dispersed particles. We acknowledge that the collection and preparation of PM may result in small differences from the freshly generated material. Nevertheless, by ensuring strict adherence to standard operating procedures and accepted practices in the field, the uniform processing steps employed for all materials provide reasonable grounds for the comparison of their relative toxicities.

Landwehr and Larcombe argue that “using immortalised human cell lines will always come second to primary human samples”. Again, we suggest that the intent of the study must determine which model is appropriate. Cell lines continue to be used in experimental research because they offer several advantages over primary cells. These include being well characterised; facilitating reproducibility across experiments and time; enabling interlaboratory comparison, etc. They are a useful screening tool. The specific cell lines used in the present study, A549 and J774, have been regularly used in toxicological investigation. While primary cells may be more applicable for the investigation of mechanisms and interindividual susceptibility, cell lines offer the potential for reproducible and high-throughput screening. We have found that J774 cells are particularly sensitive to physicochemical differences in PM, and thus appropriate for the intended purpose of this study.

We disagree with the notion that the exposure of submerged cell cultures is “less relevant” than a fully differentiated culture and exposure at the air–liquid interface. Again, we suggest that the relevant factor in determining the selection of the model is the aim of the study. If a simpler, more reproducible and more transferable model is also sensitive to the physicochemical characteristics of the particulate samples, and is predictive of in vivo effects, it is suitable for the intended purpose. Every model is just that, a model. Fully differentiated cultures exposed at the air–liquid interface, while certainly of use—especially in the context of assessing whole emissions that may be composed of gases and particles—are still only a rudimentary model of the lungs. One could dismiss the air–liquid interface exposures of differentiated cultures on the grounds that they do not encompass the complexity of cell types in the lungs, the underlying endothelial layer, the mucosal immune response, pulmonary circulation, or the interaction with the systemic response. All models are simplified versions of the real thing, each with strengths and limitations; it is how they are used in relation to a given question that defines their utility.

In contrast to Landwehr and Larcombe’s claim that our work presents a very limited array of in vivo outcomes, we tested several important in vivo toxicity outcomes in organ systems of interest. Note that the in vitro assessment of DEPs focussed on cytotoxicity and the secretion of inflammatory cytokines; accordingly, the in vivo assessment focused on a number of markers of inflammation and cardiovascular effects, often linked to inflammation. The goal was to assess the correlation between biological potency measured in vitro and in vivo effects related to the potential pathophysiological outcomes of particle exposure, and the endpoints measured were both relevant and sufficient to address the goal of the analysis. To be sure, every study selects some endpoints to examine and does not study others; ours is no different in this respect. Finally, in response to the comment regarding the volume of secondary lavage, we would like to clarify that the secondary lavage was collected using multiple volumes of 0.5 mL each, which were pooled to a total volume of 5 mL. This was carried out to increase the overall yield of lavage cells and to remove cells adhered to the lungs.

Overall, we appreciate Landwehr and Larcombe’s thorough analysis of our work; their comment, along with this response, should help to illustrate the complexity involved in the assessment of diesel exhaust emission toxicity and the need for multiple, parallel approaches to assess the toxicity of the whole exhaust versus individual emission constituents. Collectively, studies of the relative toxicity of biodiesel exhaust emissions including particulate emissions obtained through several in vitro and in vivo platforms, such as those used by us and as those described by Landwehr and Lacombe, are critical and continue to advance our understanding of the health outcomes of exposure to vehicular emissions and inform regulations.

## Data Availability

Data is contained within the original article.
